# Family Advocacy for Residents in Nursing Homes During the COVID-19 Pandemic

**DOI:** 10.1093/geroni/igab046.2928

**Published:** 2021-12-17

**Authors:** Cristina de Rosa, Yanjun Zhou, Amy Lyons, Yu-Ping Chang

**Affiliations:** University at Buffalo, Buffalo, New York, United States

## Abstract

To protect one of the most vulnerable populations from COVID-19, nursing homes enacted and enforced visiting restrictions and other measures to limit the spread of this communicable disease. Family members, many of whom are former caregivers, were suddenly cut off from nursing home residents, and struggled to maintain connection with their loved ones residing in nursing homes. The purpose of this study was to describe the experiences of family members of residents in nursing homes in advocating for residents and themselves during a time of uncertainty and many challenges. This study used a qualitative descriptive approach to conduct individual interviews. Ten family members of residents of two nursing homes in a Northeastern state were interviewed by phone or videoconference using a semi-structured guide. Interviews were transcribed verbatim and analyzed using Braun and Clarke’s Reflexive Thematic Analysis. Family members expressed concerns for the lockdown’s impact on residents’ psychosocial wellbeing in addition to the potential physical dangers of COVID-19. They explored creative means of meeting needs for information and interaction, but often felt that these efforts fell short of replicating the connectedness of in-person visits. Family members identified multiple missed opportunities for involvement in care, and voiced willingness to comply with infection prevention guidelines, such as maintaining distance, to be present with residents. Our findings indicate that family members advocated for residents’ interests to ensure quality care. Future research and policy should consider family members as a potential resource for providing care and companionship during times of crisis.

